# Psychosoziale Versorgung von Kindern und Jugendlichen im ersten Corona-Lock-Down unter Zuhilfenahme von Telefon und Online-Tools. Möglichkeiten und Grenzen

**DOI:** 10.1007/s00729-021-00184-3

**Published:** 2021-12-06

**Authors:** Michaela Haslinger, Dina Weindl, Jessica Peper-Bösenkopf, Martina Haiderer, Verena Singer, Karin Zajec

**Affiliations:** 1 Abteilung für Kinder- und Jugendpsychiatrie und Psychotherapie, Landesklinikum Baden-Mödling, Standort Hinterbrühl, Fürstenweg 8, 2371 Hinterbrühl, Österreich; 2 Institut für Psychosoziale Medizin, Psychotherapie und Kindheitsforschung, Karl Landsteiner Gesellschaft, St. Pölten, Österreich

**Keywords:** Psychosoziale Versorgung, Online-Behandlung, Telefon-Therapie, COVID-Krise, Lock-Down, Kinder, Jugendliche, Befragung, Psychosocial care, Online treatment, Telephone, Coronavirus crisis, Lockdown, Children, Adolescents, Survey, Remote Therapy

## Abstract

Die vorliegende Studie widmet sich der Analyse einer Befragung von 20 Fachkräften des Gesundheits- und Sozialbereichs, die während des ersten „Lock-Downs“ aufgrund der Covid-19-Pandemie zur Sicherstellung der psychosozialen Versorgung von Kindern und Jugendlichen Telefon und Onlinemedien benutzten. Veränderte Rahmenbedingungen, das Fehlen des persönlichen Kontakts sowie Auswirkungen auf die Behandlung (u. a. Beziehung, Inhalt, Methodik) wurden als negative Aspekte der distanzierten Behandlung beschrieben. Eine Veränderung der Privatsphäre sowie des Settings wurden sowohl als Vor- als auch Nachteil betrachtet. Als positiv wurden insbesondere Kontinuität, Flexibilität und Anonymität hervorgehoben.

## Abstract (en)

The present study is devoted to the analysis of a survey of 20 professionals from the health and social sector who used telephone and online tools to ensure psychosocial care of children and adolescents during the first lockdown due to the Covid-19 pandemic. Changed framework conditions, the lack of personal contact and effects on the treatment (including relationship, content, methodology) were described as negative aspects of distant treatment. A change in privacy and setting were seen as both an advantage and a disadvantage. Continuity, flexibility and anonymity were highlighted as positive.

## Einleitung

Als im März 2020 die erste „Corona-Welle“ Österreich erfasste und die Regierung einen pandemiebedingten „Lock-Down“ verkündete, begründete dies eine neuartige, gesamtgesellschaftliche Herausforderung. Die vorliegende explorative Studie der Karl Landsteiner Gesellschaft (Institut für Psychosoziale Medizin, Psychotherapie und Kindheitsforschung), des Kindernetzwerk Industrieviertel und der Abteilung für Kinder- und Jugendpsychiatrie und Psychotherapie, Standort Hinterbrühl des NÖ Landesklinikum Baden-Mödling (KJPP Hinterbrühl) befasst sich mit der psychosozialen Versorgung von Kindern und Jugendlichen während dieses Zeitraums. Zu diesem Zweck wurden 47 Professionist_innen unterschiedlicher Berufsgruppen, welche in Institutionen oder in freier Praxis mit Kindern und Jugendlichen im Industrieviertel (südliches Niederösterreich) arbeiten, zu ihrer Tätigkeit während des ersten „Lock-Downs“ mittels semistrukturierten Fragebogen befragt.

Ziele der Gesamtstudie waren zum Befragungszeitpunkt (1) die allgemeine Versorgungssituation im Einzugsgebiet der KJPP Hinterbrühl während dieser Ausnahmesituation zu erfassen, (2) Implikationen für weitere praktische Tätigkeiten abzuleiten, (3) ein Vergleich mit bisherigen Studien zur Online-Behandlung unter Berücksichtigung der situationsspezifischen Gegebenheiten (Weindl et al. 2020). Der aktuelle Artikel bezieht sich auf Teilergebnisse der beschriebenen Befragung. Es wird eine qualitative Analyse von 20 Interviews hinsichtlich wahrgenommener Schwierigkeiten und Grenzen sowie möglicher Vorteile einer digitalen psychosozialen Versorgung während des ersten „Lock-Downs“ vorgestellt.

Die internet- und telefonbasierte psychosoziale Versorgung wurde in den vergangenen Jahren stark diskutiert und vielfach kritisiert (Drda-Kühn et al. 2018; Hautzinger und Fuhr 2018; Noack und Weidner 2018; Reindl 2018).

Durch den ersten „Lock-Down“ in Österreich wurde eine Situation geschaffen, in der Online-Medien bzw. das Telefon vielerorts die einzige Möglichkeit darstellten, ein Behandlungs- und Beratungsangebot fortzuführen (Weindl et al. 2020). Ausgehend von der historischen Entwicklung der internetbasierten Behandlung in Österreich, in der beispielsweise Psychotherapie als (Kranken‑)Behandlung via Internet als „nicht lege artis“ (Bundesministerium für Gesundheit 2005, S. 7) verstanden wurde, handelte es sich zu Beginn der COVID-Krise wohl nicht oft um geplante Online-Interventionen mit entsprechender Vorbereitungszeit, sondern um rasch implementierte Ersatzstrukturen ohne entsprechende Rahmenbedingungen. Basierend auf diesen Gegebenheiten wurden folgende Forschungshypothesen formuliert:

- Der Entfall des persönlichen face-to-face Kontaktes führt zu deutlichen Veränderungen im Behandlungsverlauf, insbesondere in der Beziehungsgestaltung (Noack und Weidner 2018; Roesler 2017).
- Mangelnde Privatsphäre und fehlende technische Ausrüstung stellen ein Hindernis in der Inanspruchnahme von psychosozialen Versorgungsangeboten dar
- Die vermehrte Nutzung von Online-Medien während des ersten „Lock-Downs“ liefert Hinweise auf mögliche Vorteile der beschriebenen Kommunikationstools. Dies könnte in zukünftige Behandlungskonzepte einfließen (Hautzinger und Fuhr 2018).

## Methode

Über den Aufruf des Kindernetzwerk Industrieviertel wurden rund 1000 Netzwerkpartner_innen als potenzielle Studienteilnehmer_innen rekrutiert. Des Weiteren wurden 47 Mitarbeiter_innen der Kinder- und Jugendpsychiatrie Hinterbrühl kontaktiert. 60 Personen (5,45 %) erklärten sich bereit, an der Studie mitzuwirken, wobei insbesondere seitens der Mitarbeiter_innen der Kinder- und Jugendpsychiatrie ein hohes Interesse zu verzeichnen war (30 % der Interviewteilnehmer_innen). Auffällig ist, dass trotz der hohen Bereitschaft des psychiatrischen Klinikpersonals teilzunehmen, keine Ärzt_innen als Interviewpartner_innen gewonnen werden konnten. Aufgrund von Schwierigkeiten in der Terminkoordination, fehlendem Klient_innenkontakt oder hauptsächlicher Tätigkeit außerhalb des Industrieviertel Niederösterreich wurden 13 Personen von der Befragung ausgeschlossen.

Alle Interviewteilnehmer_innen wurden zu Beginn der Studie mittels ausgehändigtem Informationsblatt über die Studie informiert und gaben ihr schriftliches Einverständnis zur Teilnahme.

Insgesamt wurden 47 Interviews mit einer durchschnittlichen Dauer von über einer Stunde durch sechs Klinische- und Gesundheitspsychologinnen per Videotelefonie durchgeführt und aufgenommen. Die Interviews wurden transkribiert, auf Verständnis und Sinnhaftigkeit korrigiert und analysiert. Die Studienteilnehmer_innen wurden mittels einer Aussendung über den Studienverlauf informiert.

### Analyse

Die qualitative Auswertung erfolgte nach der Qualitativen Inhaltsanalyse nach Mayring. Durch eine induktive Kategorienbildung wurde zunächst ein primäres Kategoriensystem festgelegt und anschließend von vier Autorinnen kodiert. Die Kodes wurden im Team besprochen, abgeglichen, erweitert und weitere drei Interviews wurden von jeweils zwei Autorinnen kodiert. Das Kategoriensystem wurde nochmals diskutiert, abgeglichen und Unterkategorien wurden festgelegt. Auf Grund der Fülle der Daten, wurde in dieser Auswertungsphase der Forschungsfokus auf Schwierigkeiten/Grenzen und Vorteile einer digitalen psychosozialen Versorgung gelegt. 20 zufällig ausgewählte Interviews wurden von jeweils zwei der Autorinnen getrennt voneinander analysiert und kategorisiert und zur Sicherstellung der Interrater-Reliabilität (_K_ = 0,79) in einem zweiten Schritt kontrolliert. Unklarheiten wurden diskutiert, in das Gesamtteam getragen und bei Bedarf in das Kodierungsschemata eingearbeitet.

### Stichprobe

Die Detailergebnisse zu möglichen Schwierigkeiten und Vorteilen von digitalen Interventionen beziehen sich auf 20 (von insgesamt 47) zufällig ausgewählten Interviews. 18 Teilnehmer_innen waren weiblich und zwei männlich. Alle genannten Fachkräfte waren zwischen 25 und 59 Jahre alt. Tab. 1 zeigt die Zuordnung der Interviewpartner_innen nach Berufsgruppen.

**Table 1:** 

Berufsgruppe	Anzahl	Prozent (%)
Psychologie	7	35
Psychotherapie	4	20
Funktionelle Therapie (Ergotherapie, Physiotherapie, Logopädie)	4	20
Sozialarbeit	3	15
Jugendcoach	1	5
Kindergartenpädagogik	1	5
*Gesamt*	*20*	*100*

## Ergebnisse

Die qualitative Inhaltsanalyse zeigte, dass eine Vielzahl der Interviewteilnehmer_innen Telefonate mit Bildübertragung sowie Sprachtelefonie nutzten, um mit Kindern, Jugendlichen und Eltern zu kommunizieren. Email und Messenger-Dienste wurden hingegen seltener genannt. Die meisten Befragten äußerten klare Vorzüge hinsichtlich Video- oder Sprachtelefonie. Das tatsächlich verwendete Kommunikationsmedium wurde jedoch zumeist von den Vorlieben der Klient_innen bestimmt.

### Schwierigkeiten und Grenzen

**Figure 1:**
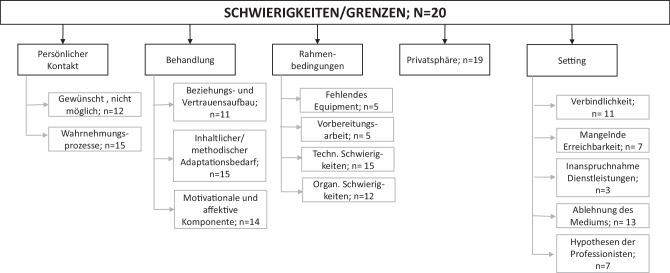

Im Zuge des Interviews wurden die Teilnehmer_innen nach Schwierigkeiten im Kontakt via Video-(telefonie), die seitens der Kinder, Jugendlichen und Eltern benannt wurden, befragt. Insbesondere interessierten zudem Grenzen der webbasierten Intervention aus Sicht der Behandler_innen (Abb. 1).

#### Persönlicher Kontakt

Als Grenze in der web- bzw. telefonbasierten Behandlung/Beratung wurde der Wunsch nach physischem Miteinander mehrfach (*n* = 12) erwähnt. *„Dass dann doch bei manchen Dingen der persönliche Kontakt unerlässlich ist“* (DiWe02, w, Altersspanne: 40–44) zeigte sich auf unterschiedlichen Ebenen: *„Mir hat auf jeden Fall […] einfach der Kontakt gefehlt. Das Gesicht dazu, die Mimik, das Miteinander in einem Raum sein, miteinander was schaffen“* (DiWe04, w, 30–34). Teilnehmer_innen beschrieben das Gefühl, dass nonverbale Bestandteile der Behandlung verloren gingen bzw. eine ganzheitliche Wahrnehmung erschwert sei (*n* = 15). Elemente, die sonst scheinbar ganz nebenbei erfasst werden, wie eine vernachlässigte Körperhygiene, konnten nur eingeschränkt bemerkt werden. Wahrgenommene Gefühle waren schwer einzuordnen:

Ich kann mich an eine Situation erinnern, wo ich gedacht habe, die Person ist jetzt traurig und weint und das habe ich dann auch nachgefragt, aber das war dann, also da habe ich dann das Feedback bekommen „Nein“. Also ich glaube es lässt sich doch besser beurteilen, wenn man vor Ort ist (JePe03, m, 40–44).

#### Behandlung

Der Kontakt zu Klient_innen, die zuvor noch nie persönlich getroffen werden konnten, stellte eine Herausforderung dar. Behandlungsverläufe, bei denen *„noch nicht so viel Beziehungsarbeit passiert ist […], wo die Behandlungsmotivation von Seiten der Jugendlichen nicht da ist“* (MaHa03, w, 30–34), wurden als besonders schwierig über Online-Medien und Telefon beschrieben.

Der Vertrauensaufbau sowie die Beziehungsarbeit wurden als problematisch geschildert (*n* = 11). Beziehungsfördernde, handelnde, eventuell auch fürsorgliche Gesten konnten nur eingeschränkt stattfinden: *„Ich kann sonst ein Glas Wasser anbieten, aber es ist etwas Anderes. Oder ich reiche das Taschentuch. Das sind kleine Dinge die gehen dann nicht“ *(MiHa03, w, 55–59).

In der bisherigen Behandlung bewährte Methoden (beispielsweise Arbeitsblätter oder kreative Möglichkeiten) konnten nicht genutzt werden.

Ich denke jetzt an eine Jugendliche. Da hast du das manchmal in der Therapie auch, dass du über das Gespräch niemand mehr erreichst und da habe ich in der Therapie andere Möglichkeiten. Da kann ich sagen, „okay wir reden jetzt nichts“, man kann aufstehen. Ich arbeite sehr gerne mit einer Sandkiste und ich kann schauen, was da möglich ist, aufzustellen, um die Blockade sichtbar zu machen oder mit einer Aufstellung oder anderem Material und das habe ich übers Telefon oder Skype nicht (MiHa03, w, 55–59).

Bei konkreten Übungen war es ausschließlich verbal möglich, einzugreifen und das Anfertigen gemeinsamer Produkte wurde deutlich erschwert. Es *„war inhaltlich einfach anstrengend, sich zu überlegen wie gehe ich es jetzt an, weil sonst habe ich schon mein Konzept, meine Materialien und dann muss man sich komplett was Neues überlegen“* (DiWe02, w, 40–44). Betont wurde in diesem Zusammenhang, dass die Spontanität, die in der Arbeit mit Kindern und Jugendlichen oft erforderlich ist, als eingeschränkt erlebt wurde.

Weil wenn ich Material vor Ort hab, kann ich es wechseln, wenn das Kind nicht zurechtkommt mit dem was ich angeboten habe. Aber per Zoom ist das nicht möglich. Also für jede Zoom-Therapie hat es eine lange Vorbereitungszeit gebraucht (JePe02, w, 55–59).

Settings wie Gruppen- oder Familientherapien stellten insbesondere zu Beginn des „Lock-Downs“ die Befragten vor große Herausforderungen.

Bemerkt wurde, dass sich der Austausch mit Personen, die unzureichend Deutsch sprechen am Telefon deutlich schwieriger gestaltete. Gleichzeitig konnten Gespräche mit Sprachmittler_innen nur eingeschränkt stattfinden.

Auch inhaltlich wurde eine Veränderung wahrgenommen – die Teilnehmer_innen berichteten, weniger konfrontativ zu arbeiten. Des Weiteren schienen Themen wie schulische oder soziale Ängste weniger präsent. Gleichzeitig konnte ein Ausprobieren in der Lebensrealität nicht erfolgen. *„Soziale Kompetenz überhaupt ist in Zeiten von Corona ein schwieriges Thema. Weil man würde ja normalerweise sagen ‚Geht’s raus‘ und ‚Trefft euch‘ […] und Verhaltensexperimente machen. Und das ist dann schon deutlicher erschwert“ *(DiWe02, w, 40–44). Seitens der Klient_innen seien bestimmte Themen explizit auf einen späteren Zeitpunkt verschoben worden, wenn eine persönliche Begegnung wieder möglich sei.

Kritisch betrachtet wurde auch die Notwendigkeit der Krisenintervention über Online-Medien: *„… wenn es um Einschätzung der Suizidalität betrifft, um akute Gefährdung […]. Am Telefon quasi unmöglich“ *(JePe01, w 25–29).

Mehrheitlich wurde die digitale Behandlung als „*anstrengend*“ beschrieben, wobei das Halten der Aufmerksamkeit bei gleichzeitig höherer Neigung zur Ablenkbarkeit betont wurde. Auch wurde der intensive und ununterbrochene Augenkontakt während der Videotelefonie als fordernd erlebt.

#### Privatsphäre

Begründet durch die Verordnung gemäß COVID-19 Maßnahmengesetz (Verordnung des Bundesministers für Soziales, Gesundheit, Pflege und Konsumentenschutz gemäß §2Z1 des COVID-19 Maßnahmengesetzes, BGBl [II] 98/2020) sowie den Appell des Bundeskanzlers zu Hause zu bleiben (Parlamentskorrespondenz 2020) verbrachte ein Großteil der Haushaltsmitglieder ihre Zeit in den eigenen vier Wänden. Dies schien deutliche Auswirkungen auf die Privatsphäre aller Beteiligten zu haben (*n* = 19). Fehlende Rückzugsmöglichkeiten in Ermangelung räumlicher Ressourcen, Probleme in der Akzeptanz der Privatsphäre anderer bzw. ein mangelndes Bewusstsein für die Notwendigkeit eines geschützten Raumes sowie Betreuungspflichten für andere Kinder im Haushalt wurden beschrieben. Trotz Bemühen, einen geschützten Rahmen herzustellen, konnte das zu Beginn einer Behandlungseinheit hergestellte Setting teils nicht aufrechterhalten werden. Die Verwendung eines digitalen Mediums (z. B. Handy), welches einen raschen Wechsel der räumlichen Gegebenheiten ermöglicht, führte zu einer Unklarheit darüber, wer sich im Behandlungsraum befindet. „*Für mich die Schwierigkeit beim Telefonieren, dass ich nicht weiß, in welchem Umfeld, in welchem Kontext ich da jetzt telefoniere. Ob die Mama danebensteht oder nicht. Das ist ganz schwierig*“ (MiHa02, m, 55–59). Insbesondere bei Klient_innen, die ohnedies Schwierigkeiten aufweisen, (eigene) Grenzen zu wahren, wurde dies beobachtet.

#### Rahmenbedingungen

Die Teilnehmer_innen beschrieben häufig die technischen und organisatorischen Rahmenbedingungen als problematisch. Fehlendes Equipment (*n* = 5), instabile Internetverbindungen oder schlechter Handyempfang, mangelnde Bild- und Tonqualität, limitierter Datenverbrauch, mangelnde Kenntnisse über die Funktionsweise von Geräten oder Videotelefonieplattformen und fehlende Kontaktdaten führten sowohl seitens der Klient_innen als auch der Fachkräfte zu Schwierigkeiten. Eine hohe Kompetenz an Selbstorganisation war gefordert:

Ich habe nur Zoom über Handy. Mein Laptop ist alt und hat keine Kamera und da habe ich jetzt von einer Freundin eine Kamera ausgeborgt und das muss ich erst ausprobieren wie das geht (JePe02, w, 55–59).

Unterbrechungen und Terminausfälle aufgrund technischer Gegebenheiten sowie die Notwendigkeit, dass die Fachkräfte auch in diesen Belangen weiterhelfen, wurden beschrieben.

Insgesamt zeigte sich auch in diesem Bereich eine deutliche Auswirkung auf das (therapeutische) Setting:

Meine gute EDV-Ausrüstung kann nicht ersetzen, wie die auf der anderen Seite ausschaut. Während ich sonst für das Setting die volle Verantwortung habe, und auch viel Kontrolle über alles was nicht im Klienten selbst drinnen ist, habe ich das beim Virtuellen gar nicht oder viel weniger. Das habe ich nur für meinen Teil. Aber ich kann nicht verhindern, dass dort die Verbindung zusammenbricht oder dort wer reinkommt (KaZa01, w, 45–49).

Auch die zeitlichen Rahmenbedingungen waren durch technische Schwierigkeiten betroffen:* „Meistens kommt es zu Verzögerungen, man braucht zehn Minuten, um das technisch hinzukriegen, dadurch ist die Stunde kürzer oder man muss überziehen.“ *(VeSi02, w, 45–49).

#### Setting

Mehrfach wurde bemerkt, dass Telefon- und Videogespräche als weniger verbindlich angesehen wurden (*n* = 11): *„Ich habe mich daran gewöhnt, dass ich erinnere dass wir jetzt Sitzung haben. Dass die Klienten vergessen. Weil sie nicht aufbrechen müssen – ist meine These“ *(KaZa01, w, 45–49). Oftmals wurde beschrieben, dass Klient_innen zum vereinbarten Zeitpunkt nicht erreichbar waren (*n* = 7). Insbesondere in der webbasierten Behandlung führte dies zu neuen Herausforderungen. Wenn Jugendliche *„… nicht einsteigen ins Zoom und man weiß dann nicht genau, woran liegts jetzt. Ist es ein technisches Problem?“ *(JePe02, w, 55–59).

Der Wegfall sehr niederschwelliger Angebote, bei denen es möglich war ohne Termin zu kommen oder Freunde zur Unterstützung mitzunehmen, schien während des „Lock-Downs“ die Hemmschwelle für die Inanspruchnahme der Behandlung zu erhöhen.

Im Kontakt mit Jugendlichen fiel auf, dass die angebotenen Kommunikationsmittel abgelehnt wurden. Wiederholt wurde in diesem Zusammenhang genannt, dass Klient_innen ihren schulischen Alltag über Online-Medien gestalten mussten und mit ihrem Freundeskreis nur darüber in Kontakt treten konnten, sodass von einer „*Übersättigung*“ ausgegangen wurde. Eine andere Hypothese lautete *„dass wir da in die digitale Welt eintreten, die ihre ist und sie uns da nicht drinnen haben wollen. Als Institution“* (DiWe03, w, 40–44).

Behandler_innen schien es schwer zu fallen, eine Balance zwischen einer Erreichbarkeit und einer Abgrenzung im Homeoffice zu erlangen. Eine Übersicht der benannten Grenzen in der psychosozialen Versorgung findet sich in Tab. 2.

**Table 2:** 

Grenzen (*N* = 20)					
Kategorien	Unterkategorien	n	Anzahl Zitate	Bedeutung	Beispiel Paraphrasen
Persönlicher Kontakt	Gewünscht, nicht möglich	12	23	Persönlicher Kontakt wird durch involvierte Personen gewünscht, ist aber nicht möglich	*Themen wurden verschoben, bis man sich wiedersieht (DiWe02); nicht die Tiefe wie bei persönl. Kontakt (MiHa04)*
	Wahrnehmungsprozesse	15	26	Auswirkungen durch Verwendung von Medien auf die Wahrnehmung	*Reaktion der Professionist:in sehen (MaHa05), nur ein Bildausschnitt sichtbar, nonverbale Kommunikation geht tw. verloren (JePe01)*
Behandlung	Beziehungs- und Vertrauensaufbau	11	21	Wie gestaltet sich der Beziehungs- und vertrauensaufbau mit digital. Kommunikationsmitteln, welche Erfahrungen werden beschrieben etc	*Man verliert den Kontakt, es bleibt an der Oberfläche (JePE11), Beziehungsaufbau ist schwieriger (DiWe02)*
	Inhaltlicher oder methodischer Adaptionsbedarf	15	35	Ursprünglich geplante Behandlungsinhalte oder Methoden mussten abgeändert werden	*Kann nicht auf die üblichen Materialen zurückgreifen (JePe11, KaZa06), gewisse Interventionen nur eingeschränkt möglich (DiWe02), es müssen andere Methoden gefunden werden (MaHa05)*
	Motivationale und affektive Komponente	14	23	(Konzentration, Langeweile etc)	*Halten der Konzentration (KaZa01), erschöpfend (KaZa01), Ablenkung ist groß (MiHa04)*
Rahmenbedingungen	Fehlendes Equipment	5	10	Kein Endgerät zur Verfügung, etc	*Digitale Ressourcen nicht gegeben (DiWe01, MiHa02)*
	Vorbereitungsarbeit	5	6	Vorbereitungen, die die Rahmenbedingungen betreffen	*Zugangsdaten für Videotelefonate versenden (JePE04), Umstellung auf neue Medien (DiWe03)*
	Technische Schwierigk	15	35	Schwierigkeiten mit techn. Ausrüstung bzw. Programmen/Apps (Handy, PC, Internet etc.) für Behandlung	*Instabile Internetverbindung. (JePe01), (KaZa01), mangelnde Kenntnisse (KaZa06)*
	Organisatorische Schwierigk	12	18	Schwierigkeiten die Behandlung ermöglichen	*Schwierigkeiten an Telefonnummern zu kommen, wo man normalerweise Kolleg:innen fragen würde (MiHa04)*
Privatsphäre	Privatsphäre	19	39	Beobachtungen, Erfahrungen, Erlebnisse, während der Behandlungen/Beratungen	*Schreierei von Eltern und Geschwistern im Hintergrund (KaZa06), Beengte Wohnverhältnisse (KaZa06, DiWe02); Sorge dass wer mithört (KaZa01), muss sehr bewusst geschaffen werden (MiHa04)*
Setting	Verbindlichkeit	11	21	Wie verbindlich zeigen sich Klient:innen?	*Wird häufiger vergessen als im persönl. Kontakt (JePe01)*
	Mangelnde Erreichbarkeit	7	8	–	*Erreicht Eltern, Klient:innen nicht, weiß nicht woran es liegt (JePe11, JePe01)*
	Inanspruchnahme von Dienstleistungen	3	6	Niederschwelligkeit vs. Hochschwelligkeit von Angeboten	*Große Hemmschwelle anzurufen, leichter anzurufen (MaHa05); man kann sich keine Unterstützung dazu holen (MaHa05)*
	Ablehnung des Mediums	13	19	–	*Telefoniert persönlich nicht gerne (DiWe02)*
	Hypothesen der Professionist:innen	7	13	Geäußerte Hypothesen zu Grenzen in der digitalen Behandlung	*Migrationshintergrund erschwert Kontakt (KaZa06); Motivation flaut nach den ersten Wochen ab (JePe01)*
Summe Zitate			312		

### Vorteile

**Figure 2:**
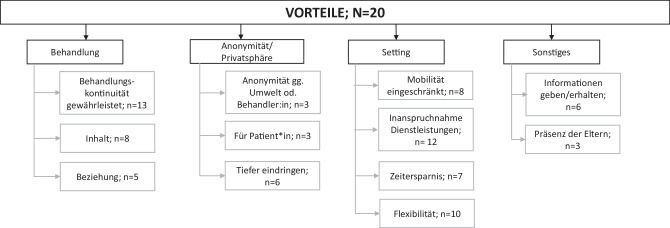

Im Rahmen der kritischen Auseinandersetzung mit einer digitalen psychosozialen Versorgung wurden sowohl seitens der Klient_innen als auch der Professionist_innen eine Reihe an Vorteilen benannt (Abb. 2).

#### Behandlung

Viele Teilnehmer_innen beschrieben, dass die Möglichkeit eines kontinuierlichen Kontakt- und Beziehungsangebots, verfügbar zu sein und somit eine weitere Versorgung zu gewährleisten, als wesentlich angesehen wurde. Dies wurde durch die Verwendung von Telefon und Online-Medien in vielen Fällen ermöglicht.

Von Einigen wurde die Möglichkeit der Krisenintervention über Online-Medien bzw. Telefon benannt: *„Gut ist Telefon in der Krise, ich glaube Krisenintervention ist ganz gut gelungen aber therapeutisch braucht es etwas Anderes“ *(MiHa03, w, 50–59).

#### Anonymität/Privatsphäre

Es wurde betont, dass (insbesondere durch die Nutzung des Telefons) ein niederschwelliger Zugang ermöglicht werden konnte. Beratungen konnten anonymer stattfinden, als dies bei einem Live-Kontakt der Fall gewesen wäre. Bei unsicheren oder sozial ängstlichen Personen entstand teilweise der Eindruck, dass diese schneller Vertrauen aufbauten und somit rascher schwierige Themen angesprochen werden konnten.

Die Möglichkeit, einen anderen Einblick in den Lebensraum der Klient:innen zu erhalten, beispielsweise indem Haustiere an Sitzungen teilnahmen, die Wohnung mit dem Handy in der Hand durchwandert wurde oder Familienmitglieder in die Kamera winkten, wurde als positiv hervorgehoben. *„Ich habe zum Beispiel über Onlinetelefonie über die Bildschirmfreigabe durch den Jugendlichen mitschauen können, wie […] der unterwegs ist und was der da wie macht […] und ich habe da Informationen bekommen, die hätte ich im Einzelgespräch nie bekommen. Plötzlich war ich in der Welt und der Jugendliche ist mir von einer ganz anderen […] Seite plötzlich begegnet, also das ist wie eine zweite Identität quasi. Also das ist, das sind schon auch Möglichkeiten, die da kommen“* (VeSi01, w, 35–39).

#### Setting

Teilnehmer_innen beschrieben, dass das Angebot von Klient_innen, mit Schwierigkeiten, außer Haus zu gehen oder mit eingeschränkter Mobilität (*n* = 8), gut genutzt wurde. Gerade im Kontakt mit Jugendlichen wurde die Finanzierung der Fahrtkosten als häufiges Problem im Alltag benannt, das durch die Nutzung von Telefon oder Video wegfiel. Im Falle von größeren Distanzen, blieben lange Fahrtzeiten erspart (*n* = 7). Dies führte wiederum zu einer größeren zeitlichen Variabilität. Hervorgehoben wurde zudem der Nutzen für Vernetzungstätigkeiten im Helfersystem.

Als weiterer Vorteil wurde die wahrgenommene zeitliche Flexibilität (*n* = 10) hervorgehoben. Termine wurden leichter an die Bedürfnisse der Klient_innen angepasst, kurzfristige Krisengespräche spontan ermöglicht, die Dauer der Termine zugunsten einer allgemein höheren Frequenz verringert oder die Stundengestaltung verändert.

#### Sonstiges

Die Option in der Krisensituation Inhalte wie Links, Ideen für Spiele und Bewegungsangebote, Kontaktadressen oder Überlegungen zur Tagesstrukturierung weiterzugeben und somit hilfreich innerhalb der Familie zu wirken wurde mehrfach als Vorteil benannt. Der oft als schwierig beschriebene Transfer in den Alltag gelang plötzlich. Es ist „*nicht anders gegangen. Es war notwendig das direkt zu Hause auszuprobieren und dann haben sie auch wirklich die Erfolge gesehen*“ (DiWe04, w, 30–34).

Die auch als Nachteil benannte Anwesenheit der Eltern führte teils zu einem besseren Verständnis der Erziehungsberechtigten für die Behandlungsziele. Diese konnten unterstützend eingreifen und sonst als abwesend erlebte Familienmitglieder in die Behandlung eingebunden werden. *„In der Beziehungsarbeit war es ein Gefühl der Verbundenheit, also mit den Eltern, mit den Kindern […]. Das war recht fein“* (JePe04, w, 25–29).

Gleichzeitig erlaubte die Nutzung mehrerer virtueller Räume auch die räumliche Trennung trotz gemeinsamer Therapie – z. B.: in strittigen Familientherapiesituationen. Eine Übersicht der benannten Vorteile in der psychosozialen Versorgung findet sich in Tab. 3.

**Table 3:** 

Vorteile (*N* = 20)					
Kategorien	Unterkategorien	n	Anzahl Zitate	Bedeutung	Beispiel Paraphrasen
Anonymität/Privatsphäre	Anonymität gg. Umwelt od. Behandler:in	3	3	Durch Behandlung über Medien ist eine größere Anonymität möglich	*Niemand bekommt mit, wohin sie gehen (MaHa05)*
	Für Patient:in	3	4	Durch Medien ist eine größere Anonymität möglich	*Man muss sein Gesicht nicht zeigen (MaHa05)*
	Tiefer Eindringen	6	11	Tiefer eindringen in das Leben der Klienten	*Einladung, das Zuhause über Video kennenzulernen (JePE02), Familieninteraktionen vor Ort wahrnehmen (KaZa01)*
Behandlung	Behandlungskontinuität gewährleistet	13	20	Stabiles Behandlungsangebot wahrnehmen und geben können	*Weiterführen der Behandlung (JePE02, DiWe02)*
	Inhalt	8	14	Gedanken und Wahrnehmungen zu Behandlungsinhalten	*Redebedarf (DiWe03), Strukturierende Maßnahmen, Lernmanagement, Emotionsregulation (VeSi02)*
	Beziehung	5	6	Positives Beziehungsgeschehen	*Gemeinsam Dasein (KaZa01), Gefühl des Verbunden Seins in Krisensituation (Jepe04)*
Setting	Inanspruchnahme von Dienstleistungen	12	29	Wahrnehmung von Angeboten auf einem niederschwelligen Niveau	*Niederschwelliger für Personen, die sich im realen Kontakt schwerer tun (JePe03)*
	Mobilität eingeschränkt	8	12	Vorteile für Personen mit eingeschränkter Mobilität	*Teilnahme wird ermöglicht (Maha05)*
	Zeitersparnis	7	9	Weniger Zeitaufwand durch digitales Angebot	*Entspannte Klient:innen weil Wegzeit wegfällt (kaZa01)*
	Flexibilität	10	20	Flexibilität im Angebot	*Uhrzeit konnte man besser an die Bedürfnisse anpassen (JePe04)*
Sonstiges	Informationen geben/erhalten	6	8	Unmittelbare Informationsweitergabe	*Eltern bekamen Informationen zu Arbeitsblättern etc. (KaZa06)* *Ideen für Spiele und Bewegungsangebote wurde gut angenommen (DiWe04)*
	Präsenz der Eltern	3	5	Verstärkte Teilhabe der Eltern	*Abwesende Eltern hereinholen (KaZa01); als Kinder unterstützend erlebt (JePe04)*
Summe Zitate			141		

## Diskussion

Die vorliegende Studie setzt sich explorativ mit Möglichkeiten und Grenzen einer psychosozialen Versorgung von Kindern und Jugendlichen unter zu Hilfenahme von Telefon und webbasierten Applikationen während des ersten „Lock-Downs“ in Österreich auseinander. Zu dieser Zeit befand sich ein Großteil der Bevölkerung zu Hause. Teile der psychosozialen Versorgung (im Industrieviertel, NÖ) wurden auf digitale und telefonische Kommunikationskanäle verlegt. Es konnte bereits in vorangegangenen Studien gezeigt werden, dass webbasierte Behandlungsangebote im Kinder- und Jugendbereich wirksam sind (Domhardt et al. 2018). Diese stellten daher während des ersten „Lock-Downs“ eine Möglichkeit einer kontinuierlichen psychosozialen Versorgung von Klient_innen dar. Eine Befragung zu Erfahrungen mit Telefon- bzw. Videokontakten während dieser Zeitperiode zeigte eine Reihe von Schwierigkeiten auf. Bezogen auf die Forschungshypothesen konnten Veränderungen im Behandlungsverlauf und in der Beziehungsgestaltung beobachtet werden. Wie von Noack und Weidner (2018) postuliert, erwies sich insbesondere der fehlende persönliche Kontakt als problematisch. Weiters zeigte sich die Notwendigkeit, dass sich die Expert_innen mit großer Flexibilität auf die neuen Umstände in der Behandlung einstellen mussten und für sie wichtige Elemente einer en-vivo Behandlung nicht eingesetzt werden konnten bzw. verloren gingen (fehlende Wahrnehmung von nonverbalen Bestandteilen und Gefühlen, problematischer Beziehungsaufbau, nicht einsetzbare beziehungsfördernde Elemente bzw. therapeutische Methoden etc.) (Schuster et al. 2018). Eine Veränderung der Behandler_innen – Klient_innenbeziehung (Roesler 2017) aber auch der Behandlung waren die Folge. Dies deckt sich mit anderen Forschungsergebnissen, welche von weniger Verbindung zwischen Behandler_innen und Patient_innen berichteten, als auch einem verringerten „Containment“ (Stewart et al. 2021). Zusätzlich zeigten sich Grenzen in der Behandlung von unzureichend Deutsch sprechenden Personen, sowohl mit Klient_innen selbst, aber auch unter zu Hilfenahme eines_einer Sprachmittler_in. Zweiteres ist überraschend, da mit Videodolmetsch in der Vergangenheit positive Erfahrungen im institutionellen Setting gemacht wurden (Kletečka-Pulker und Parrag 2018).

Als problematisch wurde auch die fehlende Privatsphäre und der fehlende „sichere“ Behandlungsraum während des „Lock-Downs“ betrachtet. Dies entspricht der Studie von Hawke et al. (2021), in welcher Jugendliche dies als Grund für eine Ablehnung der Teilnahme an virtuellen Diensten zur psychischen Gesundheit angaben. Auf der anderen Seite wurden einigen Expert_innen Einblicke in die Lebensräume der Patient_innen ermöglicht und es bleibt offen, ob dieser – durch ein digitales Medium neu geschaffene – virtuelle Raum nicht auch als Übergangsraum für psychosoziale Entwicklungsmöglichkeiten fungieren könnte (Roesler 2017). Des Weiteren, konnten teilweise bis dato eher schwer erreichbare Klient_innen aktiver in die Behandlung einbezogen werden und ein besseres Behandlungsverständnis der Eltern generiert werden.

Die Teilnehmer_innen selbst beurteilten digitale Behandlungen selbst, aber auch die technischen und organisatorischen Rahmenbedingungen als herausfordernd. Sowohl auf Behandler_innen-Seite als auch bei Klient_innen wirkte sich dies negativ auf den Behandlungsprozess und die Inanspruchnahme von psychosozialen Versorgungsangeboten aus. Technische Lösungsfindungen anstatt von Behandlungsinhalten, Unkontrollierbarkeit des Behandlungsraumes, ab- und unterbrochene Einheiten und im Laufe des „Lock-Downs“ auch eine gewisse Müdigkeit im Umgang mit digitalen Medien und bezüglich der Einhaltung von Behandlungsterminen wurden berichtet (Bailenson 2021). Auf Seiten der Expert_innen wurde der intensive Augenkontakt während der Videotelefonie als fordernd erlebt und Ergebnisse vorangeganger Forschung bestätigt (Takac et al. 2019).

Bereits vor der COVID-Pandemie konnten der telebasierten bzw. digitalen Behandlung auch positive Seiten abgewonnen werden (Hautzinger und Fuhr 2018). Die aktuelle Möglichkeit in verunsichernden Zeiten weiterhin ein Behandlungsangebot setzen zu können wurde als positiv bewertet (Stewart et al. 2021). Das digitale Format bzw. das Telefon ermöglicht eine höhere Flexibilität im Zeitmanagement aber auch in der Erreichbarkeit sowohl auf Klient_innen, als auch auf Expert_innen-Seite. Ein niederschwelliges, teilweise ressourcenschonendes Angebot kann dadurch für Klient_innen und deren Angehörige ermöglicht werden. Dies entspricht den Resultaten zu Berichten von Jugendlichen über virtuelle psychosoziale Versorgungsangeboten in aktuellen Studien (Hawke et al. 2021). Familien konnten erreicht werden, welche normalerweise auf Grund von körperlichen und/oder psychischen Einschränkungen oder räumlichen Distanzen nur schwer erreichbar waren. Aber auch die Vernetzung im Helfersystem wurde dadurch teilweise als einfacher erlebt.

## Ausblick und Limitationen

Das Studiendesign und ein erster Ausblick auf eine Ergebnisauswertung (preliminary) der hier präsentierten Studie, wurden im Rahmen der Tagung „VersorgungsNOTwendigkeit – Versorgung in der Not“ am 16.10.2020 präsentiert sowie im dazugehörigen Tagungsband (Weindl et al. 2020) veröffentlicht. Der vorliegende Artikel beschäftigt sich mit der weiterführenden Analyse und Diskussion einer größeren Datenmenge der damals skizzierten Studie.

Ressourcenbedingt konnte zum Zeitpunkt der Veröffentlichung dieses Artikels weniger als die Hälfte der erhobenen Daten analysiert werden. Eine vollständige Auswertung wäre wünschenswert, aber auf Grund des schwer überlasteten Klinikalltags und der daraus entstehenden Ressourcenknappheit derzeit kaum vorstellbar. Kritisch zu betrachten ist, dass die Erhebung während des ersten Lock-Downs, in einer Art österreichweiten Ausnahmezustandes, stattgefunden hat. Es bleibt offen, ob eine generelle Übertragbarkeit von dargestellten Ergebnissen auf webbasierte Behandlungsformen möglich ist, und sollte re-evaluiert werden.

Wie vorangegangene Studien belegen konnten, zeigen webbasierte Interventionen evidenzbasierte Wirksamkeiten (Carlbring et al. 2018; Domhardt et al. 2018). Es sollte sich jedoch, – nicht so wie beim ersten „Lock-Down“ stattgefunden – um ein ersetzendes Angebot handeln, sondern vielmehr als zusätzliche Interventionsmöglichkeit im (Klinik)alltag verstanden werden (Humer et al. 2020). Dabei sollte eine intensive Auseinandersetzung mit bereits wirksamen Interventionen und der – aufgrund des ersten „Lock-Downs“ hervorgerufenen – spezifischen Veränderungen in den einzelnen Haushalten (wie beispielsweise eine Umverteilung von Zeitressourcen, bei gleichzeitig hoher Bereitschaft zur Flexibilität; Anwesenheit aller Haushaltsmitglieder bei Ermangelung räumlicher Ressourcen; etc.) Beachtung finden. Der virtuelle Raum als zusätzliche Ressource bzw. Möglichkeit zur psychosozialen Entwicklung sollte nicht nur in Pandemie-Zeiten thematisiert werden.

Mit dem Kindernetzwerk Industrieviertel, steht der KJPP Hinterbrühl ein wertvoller Kooperationspartner gegenüber, mit dem es in den vergangenen Jahren durch viel Engagement gelungen ist, Expert_innen für Kinder und Jugendliche mit psychosozialen Schwierigkeiten zu vernetzen. Diese Ressource sollte bei einer weiterführenden Auseinandersetzung genutzt werden, um erfolgreiche Konzepte zu diskutieren und diese in einem weiteren Schritt in eine Behandlungs- bzw. Hilfeplanung mit einfließen lassen zu können. Die vorliegende Studie ist als Teil dieser Bestrebung zu sehen und zeigt, dass der Diskurs eher früher als später vorangetrieben werden sollte, um in psychisch herausfordernden Zeiten eine zeitgemäße, evidenzbasierte und professionelle Versorgung im kinder- und jugendpsychiatrischen Setting gewährleisten zu können.
